# Along the Lines of Nonadditive Entropies: *q*-Prime Numbers and *q*-Zeta Functions

**DOI:** 10.3390/e24010060

**Published:** 2021-12-28

**Authors:** Ernesto P. Borges, Takeshi Kodama, Constantino Tsallis

**Affiliations:** 1 Instituto de Física, Universidade Federal da Bahia, Salvador 40170-115, Brazil; 2 National Institute of Science and Technology of Complex Systems, Rua Xavier Sigaud 150, Rio de Janeiro 22290-180, Brazil; 3 Instituto de Física, Universidade Federal do Rio de Janeiro, Rio de Janeiro 21941-972, Brazil; 4 Instituto de Física, Campus da Praia Vermelha, Universidade Federal Fluminense and National Institute of Science and Technology for Nuclear Physics and Applications, Niterói 24210-346, Brazil; 5 Centro Brasileiro de Pesquisas Físicas, Rua Xavier Sigaud 150, Rio de Janeiro 22290-180, Brazil; 6 Santa Fe Institute, 1399 Hyde Park Road, Santa Fe, NM 87501, USA; 7 Complexity Science Hub Vienna, Josefstädter Strasse 39, 1080 Vienna, Austria

**Keywords:** nonadditive entropies, *q*-prime numbers, *q*-algebras, *q*-zeta functions

## Abstract

The rich history of prime numbers includes great names such as Euclid, who first analytically studied the prime numbers and proved that there is an infinite number of them, Euler, who introduced the function $\zeta \left(s\right)\equiv \sum_{n=1}^{\infty }n^{-s}=\prod_{p\;prime}\frac{1}{1-p^{-s}}$, Gauss, who estimated the rate at which prime numbers increase, and Riemann, who extended ζ(s) to the complex plane *z* and conjectured that all nontrivial zeros are in the R(z)=1/2 axis. The nonadditive entropy $S_{q}=k\sum_{i}p_{i}\ln_{q}\left(1/p_{i}\right)\;(q\in R;\;S_{1}=S_{BG}\equiv -k\sum_{i}p_{i}\ln p_{i}$, where BG stands for Boltzmann-Gibbs) on which nonextensive statistical mechanics is based, involves the function $\ln_{q}z\equiv \frac{z^{1-q}-1}{1-q}\;\left(\ln_{1}z=\ln z\right)$. It is already known that this function paves the way for the emergence of a *q*-generalized algebra, using *q*-numbers defined as ${\langle x\rangle }_{q}\equiv e^{\ln_{q}x}$, which recover the number *x* for q=1. The *q*-prime numbers are then defined as the *q*-natural numbers ${\langle n\rangle }_{q}\equiv e^{\ln_{q}n}\;\left(n=1,2,3,\cdots \right)$, where *n* is a prime number p=2,3,5,7,⋯ We show that, for any value of *q*, infinitely many *q*-prime numbers exist; for q≤1 they diverge for increasing prime number, whereas they converge for q>1; the standard prime numbers are recovered for q=1. For q≤1, we generalize the ζ(s) function as follows: $\zeta_{q}\left(s\right)\equiv {\langle \zeta \left(s\right)\rangle }_{q}$ (s∈R). We show that this function appears to diverge at s=1+0, ∀q. Also, we alternatively define, for q≤1, $\zeta_{q}^{\sum }\left(s\right)\equiv \sum_{n=1}^{\infty }\frac{1}{{\langle n\rangle }_{q}^{s}}=1+\frac{1}{{\langle 2\rangle }_{q}^{s}}+\cdots$ and $\zeta_{q}^{\prod }\left(s\right)\equiv \prod_{p\;prime}\frac{1}{1-{\langle p\rangle }_{q}^{-s}}=\frac{1}{1-{\langle 2\rangle }_{q}^{-s}}\frac{1}{1-{\langle 3\rangle }_{q}^{-s}}\frac{1}{1-{\langle 5\rangle }_{q}^{-s}}\cdots$, which, for q<1, generically satisfy $\zeta_{q}^{\sum }\left(s\right)<\zeta_{q}^{\prod }\left(s\right)$, in variance with the q=1 case, where of course $\zeta_{1}^{\sum }\left(s\right)=\zeta_{1}^{\prod }\left(s\right)$.

## 1. Introduction

Extending the realm of the Boltzmann-Gibbs-von Neumann-Shannon entropic functional, many measures of uncertainty have been proposed to handle complex systems and, ultimately, complexity. All of them are nonadditive, excepting the Renyi functional. Among them, a paradigmatic one is the entropy $S_{q}$ defined, with the scope of generalizing Boltzmann-Gibbs (BG) statistical mechanics, as follows [1]:

(1) $$S_{q}=k\frac{1-\sum_{i}p_{i}^{q}}{q-1}=k\sum_{i}p_{i}\ln_{q}\frac{1}{p_{i}}\;\;\left(k>0\right)$$

with $S_{1}=S_{BG}\equiv k\sum_{i}p_{i}\ln\frac{1}{p_{i}}$. The entropy $S_{q}$ is the most general one which simultaneously is composable and trace-form [2], and it has been shown to be connected to the Euler-Riemann function ζ(s) [3]. The *q*-logarithm function is defined [4] as follows

(2) $$\ln_{q}z\equiv \frac{z^{1-q}-1}{1-q}\;\;\left(\ln_{1}z=\ln z\right)\;,$$

its inverse function being

(3) $$e_{q}^{z}={\left[1+\left(1-q\right)z\right]}^{1/(1-q)}\;\left(e_{1}^{z}=e^{z}\right)\;$$

when [1+(1−q)z]≥0; otherwise it vanishes. The definitions of the *q*-logarithm and *q*-exponential functions allow consistent generalizations of algebras [5,6,7,8,9], calculus [6,8,10,11] (see also [12]) and generalized numbers [8,13].

There are different ways of defining generalized *q*-numbers connected with the pair of inverse (*q*-logarithm, *q*-exponential) functions, namely

(4) $$\begin{array}{ccc} {\langle x\rangle }_{q} & = & e^{\ln_{q}x}\;\;\;\left({\langle x\rangle }_{1}=x>0\right)\;, \end{array}$$

(5) $$\begin{array}{ccc} {}_{q}\langle x\rangle & = & e_{q}^{\ln x}\;\;\;\;\left({}_{1}\langle x\rangle =x>0\right)\;, \end{array}$$

(6) $$\begin{array}{ccc} {\left[x\right]}_{q} & = & \ln e_{q}^{x}\;\;\;\left({\left[x\right]}_{1}=x\in R\right)\;, \end{array}$$

(7) $$\begin{array}{ccc} {}_{q}\left[x\right] & = & \ln_{q}e^{x}\;\;{(}_{1}\left[x\right]=x\in R)\;. \end{array}$$

Observe that ${\langle \;{}_{q}\langle x\rangle \;\;\rangle }_{q}={}_{q}\langle \;{\langle x\rangle }_{q}\;\rangle ={\left[\;{}_{q}\left[x\right]\;\;\right]}_{q}={}_{q}\left[\;{\left[x\right]}_{q}\;\right]=x$. These four possibilities are explored in Ref. [8]. (Equations (4), (5), (6) and (7) are equivalent to Equations (11a,b) and (10a,b) of Ref. [8] respectively. The notations introduced in Equations (4) and (5) differ from those used in [8].) Other generalizations exist in the literature, also referred to as *q*-numbers [14,15]. Generalized arithmetic operations follow from each of the *q*-numbers and, consistently, there are various possibilities. The present paper will only explore one possibility for *q*-numbers, namely our Equation (4), equivalent to the iel-number Equation (11a) of [8]. For this choice, two algebras will be focused on here, namely,

(8) $${\langle x\rangle }_{q}{◯}^{q}\;{\langle y\rangle }_{q}\equiv {\langle \;x\;\circ \;y\;\rangle }_{q}$$

and

(9) $${\langle \;x{◯}_{q}\;y\;\rangle }_{q}\equiv \;{\langle x\rangle }_{q}\circ {\langle y\rangle }_{q}\;,$$

the symbol ∘ representing any of the ordinary arithmetic operators ∘∈{+,−,×,}, and ${◯}^{q}$ or ${◯}_{q}$ representing the corresponding generalized operators; naturally, ${◯}_{1}={◯}^{1}=\circ$. Possibility (8) preserves prime number factorizability, while possibility (9) does not. The algebra corresponding to Equation (8) is developed in Section 4, and it was not addressed in Ref. [8]. We use a superscript for its algebraic operators, $\circ^{q}$, a notation that was not adopted in Ref. [8]. The other algebra, corresponding to Equation (9), is presented in Section 5, and it corresponds to the oel-arithmetics addressed in Section III.D of Ref. [8], where ${}_{\left\{q\right\}}◯$ is here noted ${◯}_{q}$.

## 2. Preliminaries

Let us remind the reader, at this point, the so-called Basel problem, which focuses on the value of the series

(10) $$I_{2}=\sum_{n=1}^{\infty }\frac{1}{n^{2}}\;,$$

proposed in 1644 by Pietro Mengoli of Bologna, in contrast to the divergent harmonic series,

(11) $$I_{1}=\sum_{n=1}^{\infty }\frac{1}{n}\;.$$

This problem was intensively studied by the Bernoulli brothers from Basel almost half a century later, and became known as the Basel Problem. They proved that $I_{1}$ is divergent, but $I_{2}$ is finite and smaller than 2, but failed to obtain the exact value.

The Basel problem was solved by Euler in 1735. He also exhibited the connection with prime numbers, namely

$$I_{2}=\prod_{i=1}^{\infty }\frac{1}{1-p_{i}^{-2}}=\frac{1}{6}\pi^{2},$$

where $p_{i}$ is the *i*-th prime numbers (2,3,5,...), thus introducing for the first time the so-called Euler’s product.

(12) $$I_{n}=\prod_{i=1}^{\infty }\frac{1}{1-p_{i}^{-n}}.$$

In 1859, Riemann extended the domain of the exponent *n* to complex numbers, introducing the notation

(13) $$\begin{array}{cc} \zeta \left(s\right) & \equiv \sum_{n=1}^{\infty }\frac{1}{n^{s}} \end{array}$$

(14) $$\begin{array}{cc} \; & =\prod_{i=1}^{\infty }\frac{1}{1-p_{i}^{-s}}, \end{array}$$

so that $\zeta \left(s\right)$ is often called Riemann’s zeta function (or Euler-Riemann zeta function). By the way, Gauss made many important contributions to the field, especially the so-called prime number theorem, π(N)∼N/lnN (N→∞), where π(N) is the number of primes up to the integer *N*.

For the oncoming discussion, it is useful to remind some properties within integers which are necessary to prove

(15) $$\sum_{n=1}^{\infty }\frac{1}{n^{s}}=\prod_{i=1}^{\infty }\frac{1}{1-p_{i}^{-s}}.$$

For this, let us consider a function f(z), for z=xy, which can be written as

(16) $$f\left(z\right)=f\left(x\right)f\left(y\right).$$

The function $f\left(z\right)=\frac{1}{z^{s}}$ admits such a property. Indeed, the power law satisfies

(17) $$\begin{array}{cc} z^{a+b} & =z^{a}z^{b}, \end{array}$$

(18) $$\begin{array}{cc} z^{-a} & =\frac{1}{z^{a}}, \end{array}$$

(19) $$\begin{array}{cc} z^{ab} & ={\left(z^{a}\right)}^{b}={\left(z^{b}\right)}^{a}. \end{array}$$

Now, we know that any integer *n* can be decomposed into an unique product of prime numbers,

(20) $$n=p_{{}_{1}}^{m_{1}}p_{2}^{m_{2}}...p_{i}^{m_{i}}\cdots$$

where $m_{i}$ is the multiplicity of the prime $p_{i}$ in the product ($p_{0}=1,\;p_{1}=2,\;p_{2}=3,\;\cdots$) and they are *uniquely* determined for a given n∈N. That is, the set

(21) $$\left\{m_{1},m_{2},\cdots ,m_{i},\cdots \right\}$$

is determined for a given value of *n* so we can use the notation

(22) $$\left\{m_{1}\left(n\right),m_{2}\left(n\right),\cdots ,m_{i}\left(n\right),\cdots \right\}\;.$$

Let us focus on an interesting aspect of the primes. Taking logarithm of Equation (20), for any positive integer n∈N, we get $\ln n=\sum_{i}m{\left(n\right)}_{i}\;\ln p_{i}=\sum_{i}m{\left(n\right)}_{i}\;{\hat{e}}_{i}$ where ${\hat{e}}_{i}\equiv \ln p_{i}$. We can therefore consider the set $\left\{\ln n,\;n\in N\right\}$ as a kind of infinite dimensional vector space, whose basis vectors are $\left\{{\hat{e}}_{i},i=1,...,\right\}.$ However, rigorously, this is not a vector space, since the coefficients of the linear combination, i.e., the set of multiplicities, are only integers, and not the set of real numbers.

Note that to guarantee the uniqueness of the decomposition, the commutativity mn=nm and the associativity l(mn)=(lm)n of the product operation are essential.

Now, the sum over all positive integer values of a function f(n) satisfying Equation (16) can be written, if it converges absolutely, as

(23) $$\sum_{n=1}^{\infty }f\left(n\right)=\sum_{n=1}^{\infty }\prod_{i}^{\infty }f\left(p_{i}^{m_{i}\left(n\right)}\right)\;,$$

This comes from the fact that the direct product of the sets $\left\{p_{i},p_{i}^{2},p_{i}^{3},\cdots ,p_{i}^{m},\cdots \right\}$ for all primes is equal to the set of positive natural numbers $N^{+}\equiv \left\{1,2,3,\cdots \right\}$,

(24) $$\prod_{i=0}^{\infty }⊗\left\{p_{i},p_{i}^{2},p_{i}^{3},\cdots ,p_{i}^{m},\cdots \right\}=N^{+},$$

where ⊗ denotes the direct product. The meaning of Equation (23) can be seen more intuitively here below as

(25) $$\begin{array}{cc} \; & \begin{array}{ccccccc} \;\;\{1 & 2 & 2^{2} & 2^{3} & \cdots & 2^{m}\cdots & \}\\ ⊗\{1 & 3 & 3^{2} & 3^{3} & \cdots & 3^{m}\cdots & \}\\ ⊗\{1 & \vdots & \vdots & \vdots & \cdots & \vdots & \}\\ ⊗\{1 & p_{i} & p_{i}^{2} & p_{i}^{3} & \cdots & p_{i}^{m} & \}\\ \vdots & \vdots & \vdots & \vdots & \vdots & \vdots & \vdots \end{array}\\ \; & =\left\{1,1\cdot 2,\cdots ,p_{{}_{1}}^{m_{1}}\cdot p_{2}^{m_{2}}\cdot ...\cdot p_{i}^{m_{i}}\cdot ,\cdots \right\}=N\;. \end{array}$$

Note that the multiplicity $m_{i}\left(n\right)$ of the *i*-th prime $p_{i}$ takes all integer values if *n* runs over all natural numbers. Consistently, for a given $p_{i},$ the value of $m_{i}\left(n\right)$ runs over all nonnegative integers. That is, $m_{i}\left(n\right)$ and *i* can run over all nonnegative integers independently. Therefore, by using l(m+n)=lm+ln, we can exchange the order of the sum and the product in Equation (23):

(26) $$\sum_{n=1}^{\infty }f\left(n\right)=\prod_{i=1}^{\infty }\sum_{m=0}^{\infty }f\left(p_{i}^{m}\right)$$

if f(x) satisfies the property indicated in Equation (16).

Now, let us take the case of the Euler product,

$$f\left(n\right)=\frac{1}{n^{s}}.$$

Then the summation in *m* of Equation(26) for $p_{i}$ term is

(27) $$S\equiv \sum_{m=0}^{\infty }{\left({\left(\frac{1}{p_{i}}\right)}^{s}\right)}^{m}=1+\frac{1}{p_{i}^{s}}+{\left(\frac{1}{p_{i}^{s}}\right)}^{2}+\cdots +{\left(\frac{1}{p_{i}^{s}}\right)}^{k}+\cdots$$

which is a geometric series. By writing $r\equiv 1/p_{i}^{s}$, we obtain

(28) $$S\equiv 1+r+r^{2}+\cdots =\frac{1}{1-r}=\frac{1}{1-{\left(1/p_{i}\right)}^{s}}\;.$$

Finally, we have the Euler product form as

(29) $$\begin{array}{c} \sum_{n=1}^{\infty }\frac{1}{n^{s}}=\prod_{i=1}^{\infty }\sum_{m=0}^{\infty }{\left(\frac{1}{p_{i}^{s}}\right)}^{m}=\prod_{i=1}^{\infty }\left(\frac{1}{1-{\left(1/p_{i}\right)}^{s}}\right)\;. \end{array}$$

This connection is known as Euler’s product form. Riemann extended the domain of *s* of this function into the complex plane *z*, being since then frequently referred to as the Riemann zeta function ζ(z).

## 3. *q*-Integers

We focus on the *q*-generalized numbers ${\langle n\rangle }_{q}$, n∈N, given by Equation (4), to see how far we can conserve the essential concept of prime numbers in the set of *q*-numbers. This particular *q*-number ${\langle n\rangle }_{q}$ satisfies

(30) $$\ln\;{\langle n\rangle }_{q}=\ln_{q}n.$$

Let us consider the set of nonnegative integers N. Equation (4) defines a mapping from N to $N_{q}$, where $N_{q}$ denotes the set of all positive *q*-integers. Note that $\lim_{x\to 1}{\langle x\rangle }_{q}\to 1$ but, for other general natural numbers *n*, its *q*-partner

(31) $${\langle n\rangle }_{q}=\exp\left\{\frac{n^{1-q}-1}{1-q}\right\}$$

is not an integer, in general. This point is crucial for the question of factorization. The inverse mapping is then (see Equation (5))

(32) $$n=\exp_{q}\{\ln({\langle n\rangle }_{q})\}={}_{q}\langle \;{\langle n\rangle }_{q}\;\rangle .$$

The *q*-mapping and its inverse $N⇆N_{q}$ are one-to-one $\left[\exp_{q}\left(\ln_{q}x\right)=\ln_{q}\left(\exp_{q}\left(x\right)\right)=x,\;\forall x\in R_{+}\right]$. However, it is clear that, for q≠1, $N\neq N_{q}$.

**Figure 1:**
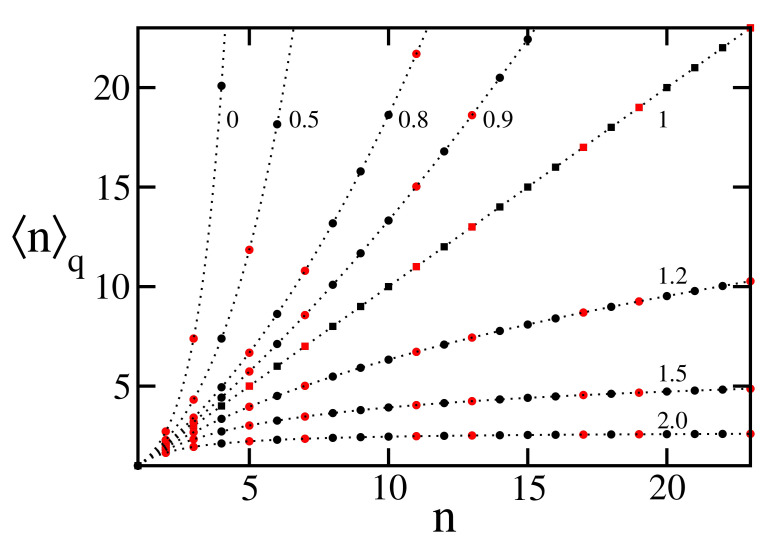
*q*-natural numbers for typical values of *q*. The *q*-prime numbers are indicated in red. The dotted lines correspond to real values for the abscissa.

For q∈(1,∞), the *q*-natural numbers satisfy $\lim_{n\to \infty }{\langle n\rangle }_{q}=e^{1/(q-1)}>1\;$. For q∈(−∞,1], the *q*-natural numbers satisfy $\lim_{n\to \infty }{\langle n\rangle }_{q}=\infty \;$. There are infinitely many *q*-prime numbers for q∈(−∞,∞). See Figure 1.

## 4. Algebra Preserving Factorizability of *q*-Integer Numbers in *q*-Prime Numbers

In order to introduce the concept of *q*-primes, we should keep the factorization concept in $N_{q}^{+}$. Thus we must first define the product operation $N_{q}$, and should be kept invariant under the *q*-mapping from $N^{+}$. Following Equation (8), let ${\langle m\rangle }_{q}{⊗}^{q}{\langle n\rangle }_{q}$ be such a product (note that here ${⊗}^{q}$ is not the direct product of sets, but this specific *q*-generalized product of *q*-numbers) between any pair of *q*-integers ${\langle m\rangle }_{q},\;{\langle n\rangle }_{q}\in N_{q}$, whose result give the *q*-transform of mn∈N, i.e., obeys the following factorizability:

(33) $${\langle m\rangle }_{q}{⊗}^{q}{\langle n\rangle }_{q}={\langle mn\rangle }_{q}.$$

We also define the following generalized summation operator ${⊕}^{q}$ in $N_{q}$ as

(34) $${\langle m\rangle }_{q}{⊕}^{q}{\langle n\rangle }_{q}={\langle m+n\rangle }_{q}.$$

The above definitions correspond to those introduced in Ref. [13] (its Equations (5) and (6); the operations defined in [13] follows the same structure of Equation (33), or, more generally, Equation (8), but with a different deformed number, namely the Heine number.).

In order that the above operations are meaningful in $N_{q}^{+}$, it is necessary that they are closed operations in $N_{q}$.

The following definitions satisfy properties (33) and (34):

(35) $${\langle m\rangle }_{q}{⊗}^{q}{\langle n\rangle }_{q}={\langle m\rangle }_{q}\;{\langle n\rangle }_{q}\;e^{\left(1-q\right)\left(\ln{\langle m\rangle }_{q}\right)\left(\ln{\langle n\rangle }_{q}\right)},$$

(36) $${\langle m\rangle }_{q}{⊕}^{q}{\langle n\rangle }_{q}=\exp\left\{\frac{{\left(m+n\right)}^{1-q}-1}{1-q}\right\}.$$

Equation (35) can be rearranged as

(37) $${\langle m\rangle }_{q}{⊗}^{q}{\langle n\rangle }_{q}={\langle m\rangle }_{q}\;{\langle n\rangle }_{q}\;e^{\ln{\langle m\rangle }_{q}\;{⊕}^{q}\;\ln{\langle n\rangle }_{q}\;-\;\ln{\langle m\rangle }_{q}\;-\;\ln{\langle n\rangle }_{q}}.$$

The result of these definitions is a *q*-integer by construction.

By construction, it is clear that this definition of the *q*-product ${⊗}^{q}$ as an operator in $N_{q}$, conserves the factorization property under the *q*-transform $n=k\;m\in N⟺\;{\langle n\rangle }_{q}={\langle k\rangle }_{q}{⊗}^{q}{\langle m\rangle }_{q}\in N_{q}.$ In addition, also by construction, the operators, ${⊗}^{q}$ and ${⊕}^{q}$ satisfy the following basic properties of algebras, valid in $N_{q}$:

- Closedness of the operation:
  (38) $$\forall {\langle m\rangle }_{q},{\langle n\rangle }_{q}\in N_{q}^{+}⟹\;{\langle m\rangle }_{q}{⊕}^{q}{\langle n\rangle }_{q}\in N_{q}^{+},\;{\langle m\rangle }_{q}{⊗}^{q}{\langle n\rangle }_{q}\in N_{q}^{+},$$
- Commutativity
  (39) $${\langle m\rangle }_{q}{⊕}^{q}{\langle n\rangle }_{q}={\langle n\rangle }_{q}{⊕}^{q}{\langle m\rangle }_{q},$$
  (40) $${\langle m\rangle }_{q}{⊗}^{q}{\langle n\rangle }_{q}={\langle n\rangle }_{q}{⊗}^{q}{\langle m\rangle }_{q},$$
- Associativity
  (41) $${\langle k\rangle }_{q}{⊕}^{q}({\langle m\rangle }_{q}{⊕}^{q}{\langle n\rangle }_{q})=({\langle k\rangle }_{q}{⊕}^{q}{\langle m\rangle }_{q}){⊕}^{q}{\langle n\rangle }_{q},$$
  (42) $${\langle k\rangle }_{q}{⊗}^{q}({\langle m\rangle }_{q}{⊗}^{q}{\langle n\rangle }_{q})=({\langle k\rangle }_{q}{⊗}^{q}{\langle m\rangle }_{q}){⊗}^{q}{\langle n\rangle }_{q},$$
- Distributivity of the product ${⊗}^{q}$ with regard to the sum ${⊕}^{q}$
  (43) $${\langle k\rangle }_{q}\;{⊗}^{q}\;({\langle m\rangle }_{q}\;{⊕}^{q}\;{\langle n\rangle }_{q})=({\langle k\rangle }_{q}\;{⊗}^{q}\;{\langle m\rangle }_{q})\;{⊕}^{q}\;({\langle k\rangle }_{q}\;{⊗}^{q}\;{\langle n\rangle }_{q}).$$
- Neutral element of the *q*-addition
  (44) $${\langle m\rangle }_{q}{⊕}^{q}{\langle 0\rangle }_{q}={\langle m\rangle }_{q}{⊕}^{q}0={\langle m\rangle }_{q}\;\;\;\left(q\geq 1\right).$$
- Neutral element of the *q*-product
  (45) $${\langle m\rangle }_{q}{⊗}^{q}{\langle 1\rangle }_{q}={\langle m\rangle }_{q}{⊗}^{q}1={\langle m\rangle }_{q}\;.$$

We show that these properties are essential to keep the nature of prime numbers for *q*-transformed integers, corresponding to Equation (20) in N.

We also define the following operations:

(46) $${\langle x\rangle }_{q}{⊘}^{q}{\langle y\rangle }_{q}={\langle x/y\rangle }_{q}$$

and

(47) $${\langle x\rangle }_{q}{⊖}^{q}{\langle y\rangle }_{q}={\langle x-y\rangle }_{q}$$

Besides, for any a∈R, we can define a *a*-power of a *q*-number by

(48) $${\langle x\rangle }_{q}{㉦}^{q}a\equiv {\langle x^{a}\rangle }_{q}$$

from what follows

(49) $$\begin{array}{cc} {\langle x\rangle }_{q}{㉦}^{q}\left(a+b\right)\; & ={\langle x^{a+b}\rangle }_{q}\\ \; & ={\langle x^{a}\rangle }_{q}\;{⊗}^{q}\;{\langle x^{b}\rangle }_{q}\\ \; & =\left({\langle x\rangle }_{q}{㉦}^{q}a\right)\;{⊗}^{q}\;\left({\langle x\rangle }_{q}{㉦}^{q}b\right), \end{array}$$

(50) $$\begin{array}{cc} {\langle x\rangle }_{q}{㉦}^{q}\left(ab\right)\; & ={\langle x^{ab}\rangle }_{q}\\ \; & ={\langle x^{a}\rangle }_{q}{㉦}^{q}b\\ \; & =\left(\;{\langle x\rangle }_{q}{㉦}^{q}a\;\right){㉦}^{q}b\\ \; & =\left(\;{\langle x\rangle }_{q}{㉦}^{q}b\;\right){㉦}^{q}a, \end{array}$$

and

(51) $$\begin{array}{cc} \left({\langle x\rangle }_{q}{㉦}^{q}s\right)\;{⊗}^{q}\;\left({\langle x\rangle }_{q}{㉦}^{q}t\right)\; & ={\left({\langle x\rangle }_{q}\right)}^{{\langle s\rangle }_{q}\;{⊕}^{q}\;{\langle t\rangle }_{q}}. \end{array}$$

As we see, for fixed *q*, this algebra is isomorphic to the standard algebra.

**Definition** **1.** *A q-integer ${\langle n\rangle }_{q}$ (i.e., an element of $N_{q}^{+}$) is called “q-prime” and written as ${\langle p\rangle }_{q}$ if it can not be written as a q-factorized form in terms of two smaller q-integers as*

(52) $${\langle n\rangle }_{q}={\langle m\rangle }_{q}{⊗}^{q}{\langle k\rangle }_{q},$$

*with ${\langle m\rangle }_{q},{\langle k\rangle }_{q}\in N_{q}^{+}$, except for the trivial factorization case, i.e., either one of ${\langle m\rangle }_{q}$ or ${\langle k\rangle }_{q}$ is the unity, ${\langle 1\rangle }_{q}$.*

It is evident from the definition of *q*-integers that all the set of *q*-primes $\left\{{\langle p_{i}\rangle }_{q},i=1,\cdots \right\}$ are *q*-partners of the prime numbers in $N^{+}$, $\left\{p_{i},i=1,...\right\}$. For any integer $n\in N^{+}$ which is not a prime, there exists the non-trivial factors, $k,m\in N^{+}$ such that n=k×m. However, from the definition of the *q*-product, Equation (33), ${\langle n\rangle }_{q}={\langle km\rangle }_{q}={\langle k\rangle }_{q}\;{⊗}^{q}\;{\langle m\rangle }_{q}$, showing that ${\langle n\rangle }_{q}$ has a non-trivial *q*-factorization and it is not a *q*-prime. As we mentioned, the *q*-correspondence between the two sets, $N^{+}$ and $N_{q}^{+}$ is one-to-one, *q*-primes in $N_{q}^{+}$ are *q*-transformations of the normal primes in $N^{+}$.

The basic property of primes is that any natural number n≥2 can be written uniquely as the products of primes $\left\{p_{i}\right\}$ as

(53) $$n=\prod_{i}p_{i}^{m_{i}\left(n\right)},$$

where

$$\left\{p_{1},p_{2},p_{3},p_{4},p_{5}\;\cdots \;\;\right\}=\left\{2,3,5,7,\;\cdots \right\}$$

is the set of primes and for a given *n*,

$$\left\{m_{1}\left(n\right),m_{2}\left(n\right),\cdots \right\}$$

is the set of multiplicities $m_{i}\left(n\right)$ of the prime $p_{i}$ is uniquely determined for given *n*.

Now, for *q*-integers, ${\langle n\rangle }_{q}\in N_{q}^{+}$, the corresponding decomposition property is valid in $N_{q}^{+}$ in terms *q*-integers with *q*-products, satisfying the properties of commutativity and distributivity. By construction, we can write for any ${\langle n\rangle }_{q}$ the *q*-prime decomposition as

(54) $${\langle n\rangle }_{q}={\prod_{i}}^{q}\;\;\left({\langle p_{i}\rangle }_{q}{㉦}^{q}m_{i}\left(n\right)\right)$$

for the sake of isomorphism of the product operations in N and $N_{q}$. The symbol $\prod^{q}$ represents the generalized product (33) of a number of terms, $\overset{n}{\underset{i}{\prod^{q}}}x_{i}\equiv x_{1}{⊗}^{q}x_{2}{⊗}^{q}\cdots {⊗}^{q}x_{n}$.

Since we have the isomorphisms of the operations of sum and product in $N^{+}$ and in $N_{q}^{+}$, we can write down the *q*-version of the Euler product:

(55) $${\sum_{{\langle n\rangle }_{q}\in N_{q}^{+}}}^{\;\;\;\;\;q}\;{\left(\frac{1}{{\langle n\rangle }_{q}}\right)}^{m\;{⊕}^{q}\;s}={\sum_{{\langle n\rangle }_{q}\in N_{q}^{+}}}^{\;\;\;\;\;q}\;\prod_{i}\;{\left({\langle p_{i}\rangle }_{q}\right)}^{m_{i}\left(n\right)},$$

with the symbol $\sum^{q}$ representing the generalized summation according to Equation (34).

Now, the multiplicity $m_{i}\left(n\right)$ of the *i*-th prime $p_{i}$ should take all integer values if *n* runs over all the integers. Inversely, for a given $p_{i}$, the value of $m_{i}\left(n\right)$ runs over all integers. That is, $m_{i}\left(n\right)$ and *i* can run over all integers independently. This comes from the fact that the direct product of the sets, $\left\{p_{i},p_{i}^{2},p_{i}^{3},\cdots ,p_{i}^{m},\cdots \right\}$, for every prime is equal to the set of natural numbers $N^{+}$ (see Equation (24)). We can therefore see that exchange the order of the sum and the product (due to the distributivity of the ordinary multiplication with regard to the ordinary addition, l(m+n)=lm+ln) in Equation (23) as

(56) $$\sum_{n\in N^{+}}f\left(n\right)=\sum_{n=1}^{\infty }\prod_{i}f\left(p_{i}^{m_{i}\left(n\right)}\right)=\prod_{i=1}\sum_{m=0}^{\infty }f\left(p_{i}^{m}\right)$$

if $f\left(x\right)$ satisfies the property Equation (16).

The definitions of the *q*-algebra introduced here is sufficient to write down the *q*-version of the Euler sum and the Euler product in *q*-representation formally as

(57) $$\zeta_{q}\left(s\right)={\sum_{\forall {\langle n\rangle }_{q}\in N_{q}}}^{\;\;\;\;\;\;q}\;\;{\langle \;{\langle 1\rangle }_{q}\;{⊘}^{q}\;\left({\langle n\rangle }_{q}^{q}s\right)\;\rangle }_{q}$$

**Figure 2:**
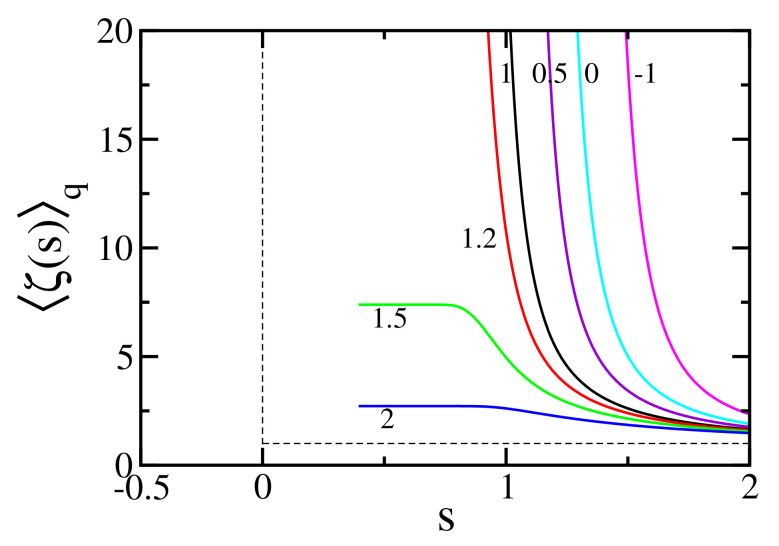
${\langle \zeta \left(s\right)\rangle }_{q}=\zeta_{q}\left(s\right)$ for typical values of *q*. These values have been calculated from Equation (59) with the first $10^{5}$ prime numbers. The *q*-number ${\langle n\rangle }_{q}$, Equation (31), with q>1 presents an upper limit, $\lim_{n\to \infty }{\langle n\rangle }_{q>1}=e^{1/(q-1)}$, and this prevents the divergence of $\zeta_{q}\left(s\right)$ for s<1.

(58) =∏qi∈N〈1〉q⊘q〈1−1/pis〉qq,

and

(59) $$\zeta_{q}\left(s\right)\equiv {\langle \zeta \left(s\right)\rangle }_{q}$$

(with $\zeta_{1}\left(s\right)\equiv \zeta \left(s\right)$). In other words, the Euler product form is preserved for all values of *q*. Figure 2 depicts $\zeta_{q}\left(s\right)$ for different values of *q*.

**Figure 3:**
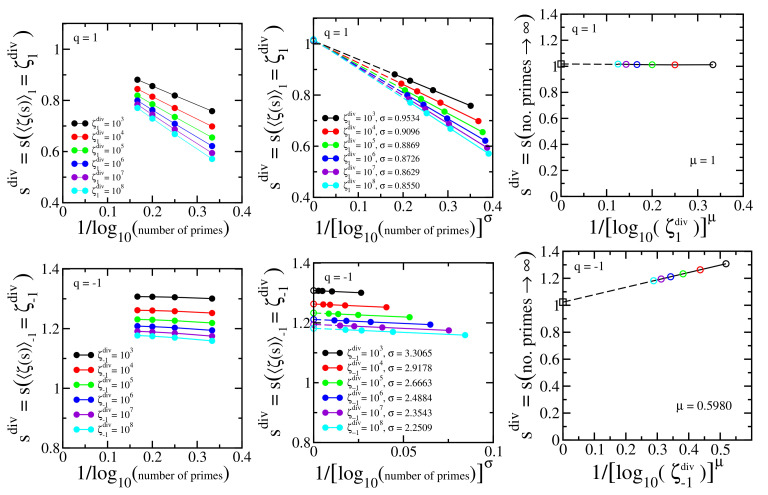
**Top left panel**: Dependence of $s^{\mathrm{div}}=s\left(\zeta_{1}^{\mathrm{div}}\right)$ with $1/\log_{10}\left(\mathrm{number}\;\mathrm{of}\;\mathrm{primes}\right)$ in Equation (14); $\zeta_{1}^{\mathrm{div}}$ is a proxy for the divergence of $\zeta_{1}\left(s\right)$. **Top middle panel**: Power-law rescaling of $s^{\mathrm{div}}=s\left(\zeta_{1}^{\mathrm{div}}\right)$ with $1/{\left[\log_{10}\left(\mathrm{number}\;\mathrm{of}\;\mathrm{primes}\right)\right]}^{\sigma }$. The curves point towards $s^{\mathrm{div}}$ with infinite number of primes (open circles); **Top right panel**: Extrapolated values of Figure 3(top middle) linearly rescaled with $1/{\left[\log_{10}\left(\zeta_{q}^{\mathrm{div}}\right)\right]}^{\mu }$ point towards the analytically exact value $s^{\mathrm{div}(\infty )}=1$ (open square) ($\lim_{\zeta_{1}^{\mathrm{div}}\to \infty }\lim_{\mathrm{number}\;\mathrm{of}\;\mathrm{primes}\to \infty }s^{\mathrm{div}}=1$) within a numerical error less than 2% for q=1. The colors of the open circles refer to the values of $\zeta_{1}^{\mathrm{div}}$ identified in Figure 3(top middle). **Bottom panels**: the same as top panels for q=−1, Equation (59).

We numerically identify the location of the divergence by fixing an arbitrary value of $\zeta_{q}\left(s\right)$ noted as $\zeta_{q}^{\mathrm{div}}$ and identify the corresponding the value of *s* (noted as $s^{\mathrm{div}}$) with increasing number of primes. The procedure is repeated with increasing values of $\zeta_{q}^{\mathrm{div}}$. The three top panels of Figure 3 illustrate the procedure for q=1, and the three bottom panels for q=−1. Each curve in Figure 3 (top left) displays the value of $s^{\mathrm{div}}$ for which $\zeta_{q}\left(s^{\mathrm{div}}\right)\equiv \zeta_{q}^{\mathrm{div}}={\langle \zeta_{1}\left(s^{\mathrm{div}}\right)\rangle }_{q}=10^{3},10^{4},\cdots ,10^{8}$ with increasing numbers of primes ($10^{3},10^{4},\cdots ,10^{6}$ primes, shown with solid circles). The representation with $1/\log_{10}\left(\mathrm{number}\;\mathrm{of}\;\mathrm{primes}\right)$ in the abscissa is not a straight line, and we empirically found that introducing a power σ (σ depends on $\zeta_{q}^{\mathrm{div}}$) as shown in Figure 3 (top middle), straight lines emerge, which can be extrapolated (dashed lines) to infinite number of primes — the open circles at the ordinate axis. These extrapolations correspond to infinite number of primes, but, nevertheless, the values of $\zeta_{q}^{\mathrm{div}}$ are still finite. Finally, the limit $\zeta_{q}^{\mathrm{div}}\to \infty$ is achieved as illustrated in Figure 3 (top right). The open circles at the ordinate axis of Figure 3 (top middle) are represented in Figure 3 (top right) for each value of $\zeta_{q}^{\mathrm{div}}$, identified with their respective colors, with the change of variables $1/{\left[\log_{10}\left(\zeta_{q}^{\mathrm{div}}\right)\right]}^{\mu }$ in the abscissa. μ is an empirical power that transforms the curves into straight lines (μ depends on *q*; μ(q=1)=1). A final extrapolation is then allowed, identifying the location of the divergence (open square). The difference $\lim_{\zeta_{1}^{\mathrm{div}}\to \infty }\lim_{\mathrm{number}\;\mathrm{of}\;\mathrm{primes}\to \infty }s^{\mathrm{div}}-1$ characterizes the numerical error. We use the same procedure for q<1, and the three bottom panels illustrate the case q=−1.

**Figure 4:**
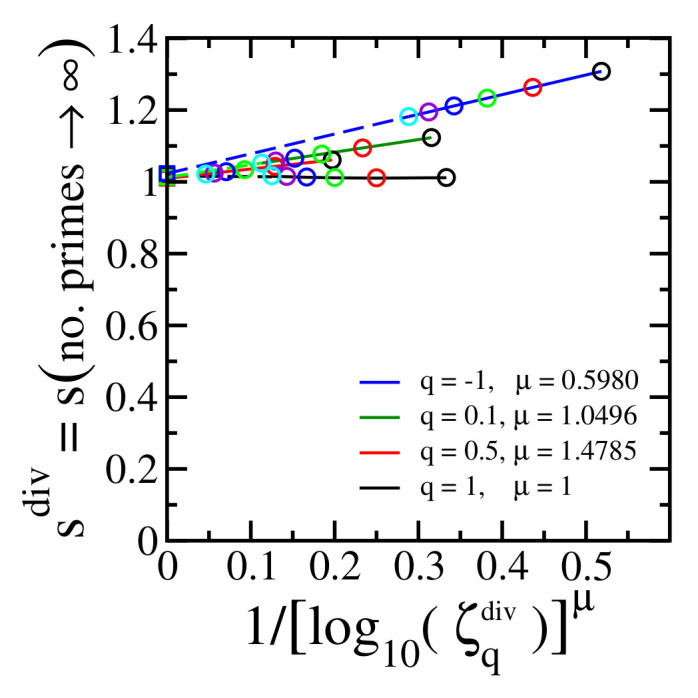
The same as Figure 3 (top right and bottom right) for typical values of q≤1. The colors of the open circles identify the value of $\zeta_{q}^{\mathrm{div}}$ as used in Figure 3 (top middle and bottom middle). The colors of the solid lines identify the value of *q* according to the legend in the present figure. The divergence of $\zeta_{q}\left(s\right)$ occurs at $s^{\mathrm{div}(\infty )}=1$ (open squares) within a numerical error less than 3% for the values of *q* that we have checked.

Figure 4 shows the final step of the procedure (see Figure 3 top right and bottom right panels) for different values of q≤1, and the maximal estimated numerical error is less than 3% for the values of *q* that we have checked. This result definitely differs from what a brief glance at Figure 2 might induce one to think.

**Figure 5:**
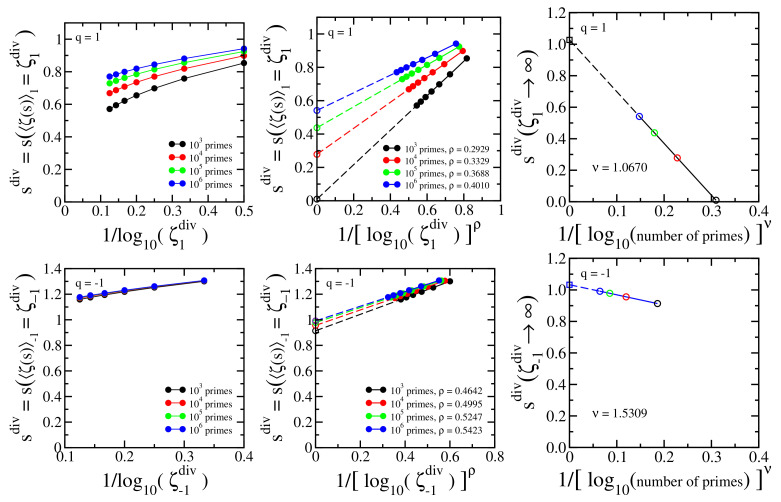
**Top left panel**: Dependence of $s^{\mathrm{div}}=s\left(\zeta_{1}^{\mathrm{div}}\right)$ with $1/\log_{10}\left(\zeta_{1}^{\mathrm{div}}\right)$ in Equation (14); **Top middle panel**: Power-law rescaling of $s^{\mathrm{div}}=s\left(\zeta_{1}^{\mathrm{div}}\right)$ with $1/{\left[\log_{10}\left(\zeta_{1}^{\mathrm{div}}\right)\right]}^{\rho }$. The curves point towards $s^{\mathrm{div}}$ with $\zeta_{q}^{\mathrm{div}}\to \infty$ (open circles); **Top right panel**: Extrapolated values of Figure 5 (top middle) linearly rescaled with $1/{\left[\log_{10}\left(\mathrm{number}\;\mathrm{of}\;\mathrm{primes}\right)\right]}^{\nu }$ point towards $s^{\mathrm{div}(\infty )}=1$ (open square) ($\lim_{\mathrm{number}\;\mathrm{of}\;\mathrm{primes}\to \infty }\lim_{\zeta_{1}^{\mathrm{div}}\to \infty }s^{\mathrm{div}}=1$) within a numerical error less than 3%. The colors of the open circles refer to the number of primes identified in Figure 5 (top middle). **Bottom panels**: the same as top panels with q=−1, Equation (59).

**Figure 6:**
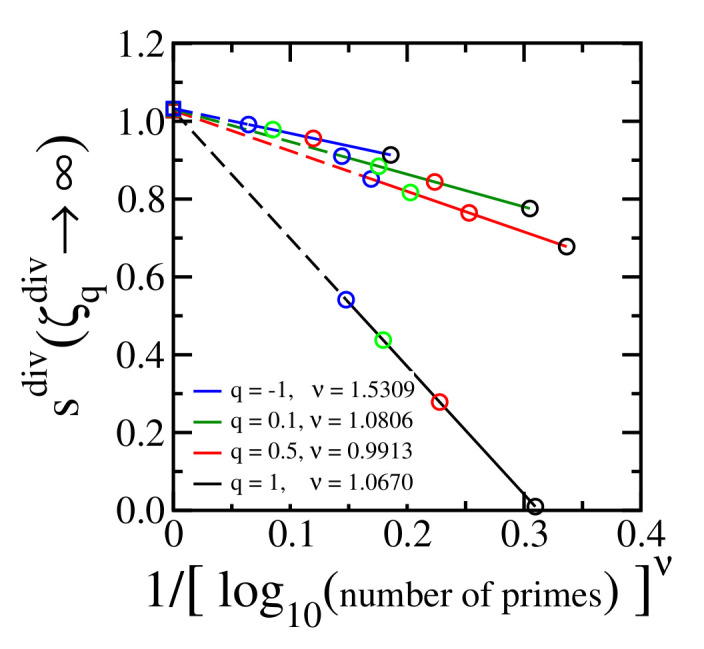
The same as Figure 5 (top right and bottom right) for typical values of q≤1. The colors of the open circles identify the number of primes as used in Figure 5 (top middle and bottom middle). The colors of the solid lines identify the value of *q* according to the legend in the present figure. The divergence of $\zeta_{q}\left(s\right)$ occurs at $s^{\mathrm{div}(\infty )}=1$ (open squares) within a numerical error less than 4% for the values of *q* that we have checked.

The numerical procedure can be taken in the inverse order, taking $\zeta_{q}^{\mathrm{div}}\to \infty$ as the first step, and then taking increasing number of primes: see Figure 5 and Figure 6. Each curve in Figure 5 (top left and bottom left) displays the value of $s^{\mathrm{div}}$ calculated with the same number of primes in Equation (59) ($10^{3},10^{4},10^{5},10^{6}$ primes) as a function of $1/\log_{10}\zeta_{q}^{\mathrm{div}}$. Here, similarly to Figure 3 (top left and bottom left), the curves are not straight lines, so they can hardly be extrapolated. The empirical power-law rescaling shown in Figure 5 (top middle and bottom middle) indicates that $s^{\mathrm{div}}$ linearly scales with $1/{\left[\log_{10}\zeta_{q}^{\mathrm{div}}\right]}^{\rho }$ (ρ depends on the number of primes), and the generated straight lines point to the corresponding values of $s^{\mathrm{div}}$ with infinite number of primes in the $\zeta_{q}\left(s\right)$ function (open circles of the top middle and bottom middle panels). These extrapolated values are rescaled according to a power-law shown in Figure 5 (top right and bottom right), with the empirical power ν depending on *q*.

Figure 6 is equivalent to top right and bottom right panels of Figure 5 for different values of q≤1. All these cases indicate $\lim_{\mathrm{number}\;\mathrm{of}\;\mathrm{primes}\to \infty }\lim_{\zeta_{q}^{\mathrm{div}}\to \infty }s^{\mathrm{div}}=1$ within a numerical error less than 4%

The empirical powers (σ, ρ, μ, ν) have been estimated by fitting a parabola $y=a+bx+cx^{2}$ to the corresponding curve, *x* is the variable of the abscissa of the corresponding middle and right panels, $y=s^{\mathrm{div}}$, and the fitting value of the power is that for which c≈0, estimated with four digits for the power parameter. The coefficient *a* of the fitting of the parabola is the extrapolated value of the corresponding curve (open circles of the top middle and bottom middle panels of Figure 3 and Figure 5, open squares of the top right and bottom right panels of Figure 3 and Figure 5 and open squares of Figure 4 and Figure 6).

Similar behavior is expected for $\zeta_{q}\left(s\right)$ evaluated with the version with summations, Equation (57).

## 5. Algebra Violating Factorizability of *q*-Integer Numbers in *q*-Prime Numbers

The *q*-product corresponding to Equation (9),

(60) $${\langle x{⊗}_{q}y\rangle }_{q}={\langle x\rangle }_{q}\;{\langle y\rangle }_{q},$$

is defined as (Equation (7) of [6], Equation (48) of [8])

(61) $$x\;{⊗}_{q}\;y\equiv {\left[x^{1-q}+y^{1-q}-1\right]}^{\frac{1}{1-q}}\;\;\;\;\left(x\geq 0,\;\;y\geq 0;\;x{⊗}_{1}y=xy\right)$$

or, equivalently,

(62) $$x{⊗}_{q}y\equiv e_{q}^{\ln_{q}x+\ln_{q}y}\;,$$

and the *q*-sum is defined as (Equation (A19) of [8])

(63) $$\begin{array}{ccc} x{⊕}_{q}y & \equiv & e_{q}^{\ln\;[e^{\ln_{q}x}+e^{\ln_{q}y}]}\\ & = & {\left\{1+\left(1-q\right)\ln[e^{\frac{x^{1-q}-1}{1-q}}+e^{\frac{y^{1-q}-1}{1-q}}]\right\}}^{1/(1-q)}. \end{array}$$

If q≠1, ${\langle x\rangle }_{q}{\langle y\rangle }_{q}\neq {\langle x\;y\rangle }_{q}$. Equation (63) with the symbol ${⊕}_{q}$ is equivalent to Equations (44) and (A19) of [8] with the symbol ${}_{\left\{q\right\}}⊕$, called oel-addition. (The notation ${⊕}_{q}$ adopted in Equation (63) was used as Equation (4) of [6] with a *different* meaning than here, namely $x{⊕}_{q}y\equiv x+y+\left(1-q\right)xy$, which is usually referred to as *q*-sum. x+y+(1−q)xy is denoted in Ref. [8] (Equation (25)) with the symbol $x\;{}_{\left[q\right]}\;⊕y$ and is called ole-addition.)

The properties (38)–(45) also hold for the ${}_{q}$ operations (see [8]).

(64) ζq∑′(s)≡∑qn=1∞1ns=1⊕q12s⊕q13s⊕q⋯

and

(65) ζq∏′(s)≡∏qpprime11−p−s=11−2−s⊗q11−3−s⊗q11−5−s⊗q⋯

In the next Section we present details on a specific generalization.

## 6. *q*-Generalizations of the ζ(*s*) Function Directly Stemming from *q*-Numbers

**Figure 7:**
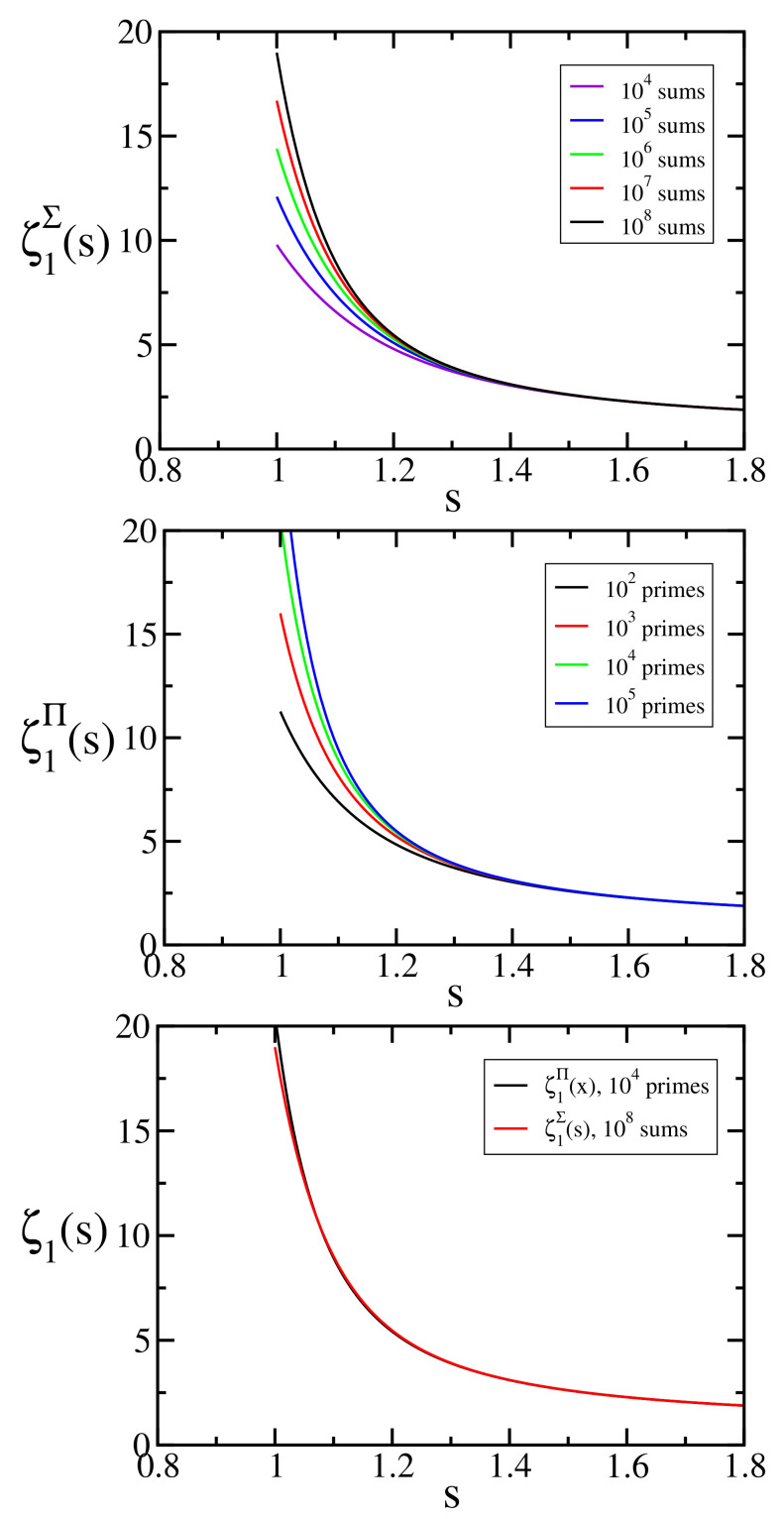
Influence on $\zeta_{1}^{\sum }\left(x\right)=\zeta_{1}^{\prod }\left(x\right)=\zeta \left(x\right)$ of the number of terms in the sum and in the product, where we have used respectively Equation (66) and (67).

**Figure 8:**
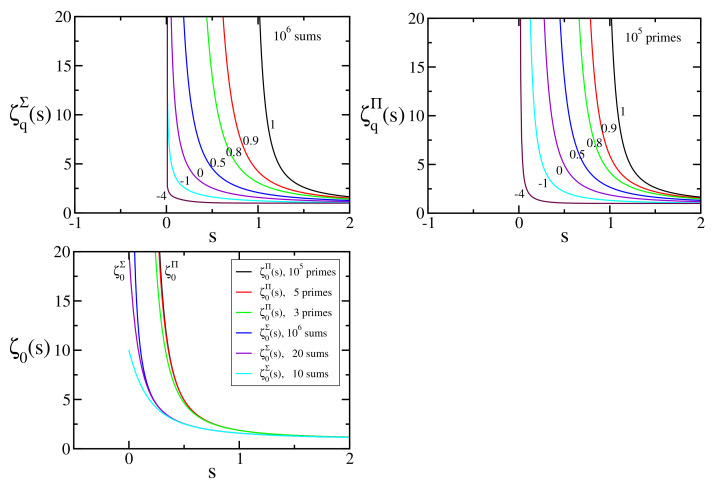
$\zeta_{q}^{\sum }\left(s\right)$ and $\zeta_{q}^{\prod }\left(s\right)$ for typical values of q≤1.

We define the following *q*-generalizations of the Riemann ζ function:

(66) $$\zeta_{q}^{\sum }\left(s\right)\equiv \sum_{n=1}^{\infty }\frac{1}{{\langle n\rangle }_{q}^{s}}=1+\frac{1}{{\langle 2\rangle }_{q}^{s}}+\frac{1}{{\langle 3\rangle }_{q}^{s}}+\cdots \;\left(s\in R\right)$$

and

(67) $$\begin{array}{ccc} \zeta_{q}^{\prod }\left(s\right) & \equiv & \prod_{p\;prime}\frac{1}{1-{\langle p\rangle }_{q}^{-s}}\\ & = & \frac{1}{1-{\langle 2\rangle }_{q}^{-s}}\frac{1}{1-{\langle 3\rangle }_{q}^{-s}}\frac{1}{1-{\langle 5\rangle }_{q}^{-s}}\cdots \;, \end{array}$$

where ${\langle n\rangle }_{q}$ is the *q*-number defined in Equation (4); see Figure 7 and Figure 8.

Other possibilities may naturally be considered for generalizing ζ(s), for instance

(68) $$\zeta_{q}^{\sum^{\prime \prime }}\left(s\right)\equiv \sum_{n=1}^{\infty }{\langle \frac{1}{n^{s}}\rangle }_{q}=1+{\langle \frac{1}{2^{s}}\rangle }_{q}+{\langle \frac{1}{3^{s}}\rangle }_{q}+\cdots$$

and

(69) $$\begin{array}{ccc} \zeta_{q}^{\prod^{\prime \prime }}\left(s\right) & \equiv & \prod_{p\;prime}{\langle \frac{1}{1-p^{-s}}\rangle }_{q}\\ & = & {\langle \frac{1}{1-2^{-s}}\rangle }_{q}{\langle \frac{1}{1-3^{-s}}\rangle }_{q}{\langle \frac{1}{1-5^{-s}}\rangle }_{q}\cdots \end{array}$$

Let us however clarify that it is not the scope of the present paper to systematically study all such possibilities.

## 7. Final Remarks

**Figure 9:**
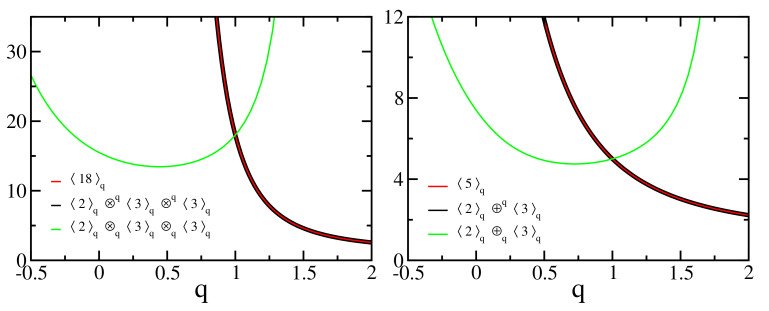
**Left panel**: Factorization is preserved through the generalized product defined by (33), illustrated with ${\langle 2\rangle }_{q}{⊗}^{q}{\langle 3\rangle }_{q}{⊗}^{q}{\langle 3\rangle }_{q}$ for different values of *q* (black curve); the generalized product defined by (62), ${\langle 2\rangle }_{q}{⊗}_{q}{\langle 3\rangle }_{q}{⊗}_{q}{\langle 3\rangle }_{q}$, does not preserve factorization (green curve). The red curve is the corresponding *q*-number, Equation (4). **Right panel**: instance of the *q*-sum of *q*-numbers, with the *q*-sums given by (34), ${\langle 2\rangle }_{q}{⊕}^{q}{\langle 3\rangle }_{q}$, (black curve) and (63), ${\langle 2\rangle }_{q}{⊕}_{q}{\langle 3\rangle }_{q}$, (green curve). The red curve is the corresponding *q*-number, Equation (4). Notice that there exists a nontrivial value of q≠1 for which ${\langle 2\rangle }_{q}{⊗}_{q}{\langle 3\rangle }_{q}{⊗}_{q}{\langle 3\rangle }_{q}=18$ and, similarly, ${\langle 2\rangle }_{q}{⊕}_{q}{\langle 3\rangle }_{q}=5$.

Let us now illustrate the two algebraic approaches focused on in the present paper (see Figure 9):

(70) $${\langle 18\rangle }_{q}={\langle 2\rangle }_{q}{⊗}^{q}{\langle 3\rangle }_{q}{⊗}^{q}{\langle 3\rangle }_{q}={\langle 2\rangle }_{q}{⊗}^{q}\left({\langle 3\rangle }_{q}{㉦}^{q}2\right)\;\;\;\left(\forall q\right)$$

whereas

(71) $${\langle 18\rangle }_{q}\neq {\langle 2\rangle }_{q}{⊗}_{q}{\langle 3\rangle }_{q}{⊗}_{q}{\langle 3\rangle }_{q}\neq {\langle 2\rangle }_{q}{⊗}_{q}\left({\langle 3\rangle }_{q}{㉦}^{q}2\right)\;\;\;\left(\forall q\neq 1\right).$$

Analogously we have

(72) $${\langle 5\rangle }_{q}={\langle 2\rangle }_{q}{⊕}^{q}{\langle 3\rangle }_{q}\;\;\;\left(\forall q\right)$$

whereas

(73) $${\langle 5\rangle }_{q}\neq {\langle 2\rangle }_{q}{⊕}_{q}{\langle 3\rangle }_{q}\;\;\;\left(\forall q\neq 1\right).$$

The algebra preserving the factorizability of *q*-integer numbers into *q*-prime numbers (see equality (70)) achieves this remarkable property essentially because it is isomorphic to the usual prime numbers. On the other hand, precisely because of that, it is unable to properly *q*-generalize the concept of a vectorial space in terms of nonlinearity. In contrast, the algebra which violates the factorizability of *q*-integer numbers into *q*-prime numbers (see inequality (71)), or some similar algebra, emerges as a possible path for achieving the concept of nonlinear vector spaces, which has the potential of uncountable applications in theoretical chemistry and elsewhere.

Since the inequality relation between *q*-primes remains the same as that for q=1, it is plausible that the nontrivial zeros in the analytic extension behaves similarly. More precisely, it might well be that, by extending $\zeta_{q}\left(s\right)$, $\zeta_{q}^{\sum }\left(s\right)$ and $\zeta_{q}^{\prod }\left(s\right)$ to the complex plane *z*, all nontrivial zeros belong to specific single continuous curves, $R\left(z\right)=f_{q}\left(I\left(z\right)\right)$, thus *q*-generalizing the q=1 Riemann’s 1859 celebrated conjecture R(z)=1/2.

We have here explored generalizations of the ζ(s) function based on a specific type of *q*-number, Equation (4), and two associated generalized algebras (Section 4 and Section 5). Three additional forms of *q*-generalized numbers, Equations (5), (6) and (7), are identified in Ref. [8]. To each of these *q*-numbers, we can associate two consistently generalized algebras, one of them violating the factorizability in prime numbers (see Ref. [8]), the other one following along the lines of Section 4. Similarly, various other generalizations of the ζ(s) function may of course be developed. Naturally, the extension of the present *q*-generalized ζ(s) functions to complex *z* surely is interesting, but does not belong to the aim of the present effort. In any case, the intriguing fact that various infinities appear to linearly scale with negative powers of logarithms might indicate some general tendencies.

It is well known that both random matrices and quantum chaos (classically corresponding to strong chaos, i.e., *positive* maximal Lyapunov exponent) [16,17,18,19,20] are related to the Riemann ζ-function and prime numbers. On the other hand, both random matrices and strong chaos have been conveniently *q*-generalized, in [21,22,23,24] respectively. These facts open the door for possible applications of the present *q*-generalizations of prime numbers and of the ζ-function to *q*-random matrices and to weak chaos (classically corresponding to *vanishing* maximal Lyapunov exponent, which recovers strong chaos in the q→1 limit). Moreover, connections of the present developments within the realm of the theory of numbers, or, more specifically, the theory of prime numbers, remain, at this stage, out of our scope. Further work along these lines would naturally be very welcome.

## Data Availability

Data sharing not applicable.

## References

[1] Tsallis C. (1988). Possible generalization of Boltzmann-Gibbs statistics. J. Stat. Phys..

[2] Enciso A., Tempesta P. (2017). Uniqueness and characterization theorems for generalized entropies. J. Stat. Mech..

[3] Tempesta P. (2011). Group entropies, correlation laws, and zeta functions. Phys. Rev. E.

[4] Tsallis C. (1994). What are the numbers that experiments provide?. Quim. Nova.

[5] Nivanen L., Méhauté A.L., Wang Q.A. (2003). Generalized algebra within a nonextensive statistics. Rep. Math. Phys..

[6] Borges E.P. (2004). A possible deformed algebra and calculus inspired in nonextensive thermostatistics. Phys. A.

[7] Nivanen L., Wang Q.A., Méhauté A.L., Kaabouchi A.E., Basillais P., Donati J.D., Lacroix A., Paulet J., Perriau S., Chuisse S.S. (2009). Hierarchical structure of operations defined in nonextensive algebra. Rep. Math. Phys..

[8] Borges E.P., da Costa B.G. (2021). Deformed mathematical objects stemming from the *q*-logarithm function. arXiv.

[9] Gomez I.S., Borges E.P. (2021). Algebraic structures and position-dependent mass Schrödinger equation from group entropy theory. Lett. Math. Phys..

[10] Kalogeropoulos N. (2005). Algebra and calculus for Tsallis thermo-statistics. Phys. A.

[11] Nobre F.D., Rego-Monteiro M.A., Tsallis C. (2011). Nonlinear Relativistic and Quantum Equations with a Common Type of Solution. Phys. Rev. Lett..

[12] Czachor M. (2020). Unifying Aspects of Generalized Calculus. Entropy.

[13] Lobao T.C.P., Cardoso P.G.S., Pinho S.T.R., Borges E.P. (2009). Some properties of deformed *q*-numbers. Braz. J. Phys.

[14] Haran M.J.S. (2001). The Mysteries of the Real Prime.

[15] Kac V., Cheung P. (2002). Quantum Calculus.

[16] Berry M.V., Seligman T.H., Nishioka H. (1986). Riemann’s zeta function: A model for quantum chaos?. Quantum Chaos and Statistical Nuclear Physics.

[17] Berry M.V., Keating J.P. (1999). The Riemann Zeros and Eigenvalue Asymptotics. SIAM Rev..

[18] Keating J.P., Snaith N.C. (2000). Random matrix theory and *ζ*(1/2 + *i**t*). Commun. Math. Phys..

[19] Firk F.W.K., Miller S.J. (2009). Nuclei, primes and the random matrix connection. Symmetry.

[20] Kriecherbauer T., Marklof J., Soshnikov A. (2001). Random matrices and quantum chaos. Proc. Natl. Acad. Sci. USA.

[21] Toscano F., Vallejos R.O., Tsallis C. (2004). Random matrix ensembles from nonextensive entropy. Phys. Rev. E.

[22] Weinstein Y.S., Lloyd S., Tsallis C. (2002). Border between between regular and chaotic quantum dynamics. Phys. Rev. Lett..

[23] Weinstein Y.S., Tsallis C., Lloyd S., Elze H.-T. (2004). On the emergence of nonextensivity at the edge of quantum chaos. Decoherence and Entropy in Complex Systems.

[24] Queiros S.M.D., Tsallis C., Beck C., Benedek G., Rapisarda A., Tsallis C. (2005). Edge of chaos of the classical kicked top map: Sensitivity to initial conditions. Complexity, Metastability and Nonextensivity, Proceedings of the 31st Workshop of the International School of Solid State Physics, Erice, Italy, 20–26 July 2004.

